# The Physical Factor in Emigration

**Published:** 1921-01-22

**Authors:** 


### The Physical Factor in Emigration.

Within the past few weeks the writer has received a number of applications for assistance from ex-soldiers suffering from one form of disability or another who are anxious to emigrate to Canada. It is rather unfortunate, perhaps, that the hopes of these men and of others should have been directed towards the Dominions through the casual advice offered some time ago by the Prime Minister to the surplus population of these isles to emigrate. It is well that there should be no misunderstanding on this point. There is, as far as we can see, no hope for any Englishman who is without capital and unfit in the prospect of emigration.

A very clear account of the situation in Canada has been sent to the *Daily News* by its Canadian correspondent, who points out that Canada is at present faced with a serious unemployment problem of its own.

The situation in British Columbia, whose climatic charms induced a large number of soldiers who enlisted from elsewhere to take their discharge and settle within its bounds, is the most serious of all; and, in view of the difficult conditions, the Immigration Department has decreed that all save a few limited classes of immigrants, immediately capable of farm work, are to be barred from Canada for three months from the commencement of the present year unless they can show themselves possessed of $250, which at the present rate of exchange is equivalent to £60. The true history of British emigration to Canada in the last twenty years is certainly discouraging, to say the least. The cold fact is, this correspondent reminds us, that an unfortunately large number of the people who sailed from English ports with high hopes for a happy and prosperous life in Canada failed to realise their dreams. Their poor physique and their early environment were serious obstacles to success in a new country. Many drifted back to England, and not a few moved southward to the industrial centres of the United States.

And then comes rather a nasty knock at the Old Country. Before there can be any success for "grandiose schemes of colonisation" started in English soil there must be, we are informed, "wholesale measures of social reform in England which will improve the health and physique of future emigrants." It might have been less bluntly said, but it is not far from the unpalatable truth.

